# Euclidean-Lorentzian Dichotomy and Algebraic Causality in Finite Ring Continuum

**DOI:** 10.3390/e27111098

**Published:** 2025-10-24

**Authors:** Yosef Akhtman

**Affiliations:** 1 Gamma Earth Sàrl, 1162 Morges, Switzerland; ya@gamma.earth; 2 Faculty of Space Technologies, AGH University of Krakow, 30-059 Krakow, Poland

**Keywords:** finite fields, quadratic forms, finite ring cosmology, algebraic causality, relativistic algebra, symmetry classes, discrete spacetime, relational physics

## Abstract

We present a concise and self-contained extension of the Finite Ring Continuum (FRC) program, showing that symmetry-complete prime shells $F_{p}$ with p=4t+1 exhibit a fundamental Euclidean-Lorentzian dichotomy. A genuine Lorentzian quadratic form cannot be realized within a single space-like prime shell $F_{p}$, since to split time from space one requires a time coefficient $c^{2}$ in the nonsquare class of $F_{p}^{\times }$, but then $c\notin F_{p}$. An explicit finite-field Lorentz transformation is subsequently derived that preserves the Minkowski form and generates a finite orthogonal group $O(Q_{\nu },F_{p^{2}})$ of split type (Witt index 1). These results demonstrate that the essential algebraic features of special relativity—the invariant interval and Lorentz symmetry—emerge naturally within finite-field arithmetic, thereby establishing an intrinsic relativistic algebra within FRC. Finally, this dichotomy implies the algebraic origin of causality: Euclidean invariants reside within a space-like shell $F_{p}$, while Lorentzian structure and causal separation arise in its quadratic spacetime extension $F_{p^{2}}$.

## 1. Introduction

**Figure 1:**
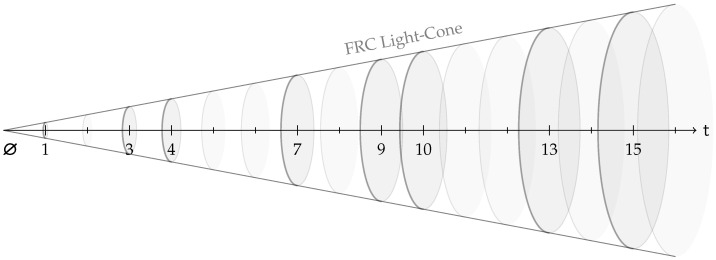
Schematic of the first 16 counts of the chronon parameter t and the corresponding arithmetic symmetry shells of the order q=4t+1. The prime shells $S_{p}$ formed by the symmetry-complete fields $F_{p}$, where p is prime, are emphasized.

The present note is framed within the broader program of the Finite Ring Continuum (FRC) [1]. The following physical interpretation of FRC is inspired by, and conceptually aligned with, the finite and relational perspectives articulated by Lev [2] and Smolin [3]. Within this framework, the physical universe is modelled as an ensemble of finite arithmetic symmetry shells $U_{t}$ formed by a succession of finite algebraic rings $Z_{q}$, with q=4t+1 and t being a time-like discrete radial chronon parameter, as illustrated in Figure 1. Each shell supports three fundamental arithmetic actions—translation $T_{a}$, scaling $S_{m}$, and powering $P_{\epsilon }$—which are interpreted as rotational symmetries and generate a (1,3)-dimensional symbolic symmetry space $U={⋃}_{t}U_{t}$.

For the specific values of t, such that p=4t+1 is prime, the resultant geometric structure manifests itself as a combinatorial 2-sphere $S_{p}$ embedded in a (1,3)-D symmetry space U, with meridians and latitudes corresponding to additive and multiplicative rotational symmetries. Physical observables are identified not with individual residues of $F_{p}$ but with stable symmetry classes (quadratic residues, Klein-four orbits, etc.). In the cosmological reading, the linear succession of shells $U_{t}$ models the passage of cosmic time, while the complex of internal symmetries $S_{q}$ of each shell encode the local laws of physics. Within this setting, the present paper isolates a key phenomenon: Lorentzian signature cannot be realized internally to a space-like prime shell $S_{p}$; it can only be realized through its space-time quadratic extension $F_{p^{2}}$ of the shell $S_{p}$. We interpret this purely emergent phenomenon as the *algebraic origin of causality*.

From the perspective of the global FRC timeline, each shell $S_{q}$ constitutes an accumulation of structure, symmetry, and thus information as the chronon parameter t advances. Yet from the perspective of a finite observer with a fixed information horizon, the growing complexity of the ambient symmetry space appears as an irreversible build-up of entropy. This observer-relative distinction between absolute information and perceived entropy provides a natural bridge to the Second Law of Thermodynamics.

**Figure 2:**
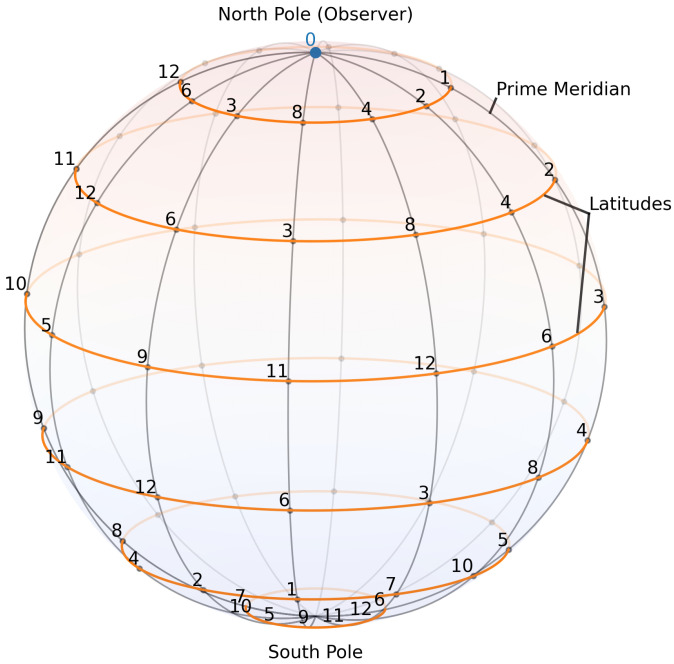
State diagram for framed finite field $F_{13}\left(3;0,1,2\right)$ as a 2D spheroid in (1,3)-D symmetry space U combining the additive symmetry along the meridians $M_{n}\left(a\right)$, as well as multiplicative symmetry along the latitudes $L_{a}\left(m\right)$ for multiplicative generator g=2.

More specifically, a prime shell $S_{p}$ of order p=4t+1 is formed by a symmetry-complete finite field $F_{p}$ [1] with fourth roots of unity {1,i,−1,−i} and a 3D rotational structure encoded by additive and multiplicative actions; the ambient symmetry space is $U={⋃}_{t}U_{t}$, and a 2D orbital complex $S_{p}\subset U$ is built by the meridians $M_{n}\left(a\right)=ag^{n}$ and latitudes $L_{a}\left(m\right)=ag^{m}$, where *g* is a primitive root of $F_{p}$, while the power-map parameter ϵ is frozen as depicted in Figure 2.

A persistent question is: how can one realize a Lorentzian metric (split signature) on such a shell [4]? We prove that, algebraically, one cannot do this within $F_{p}$ for p≡1(mod4). The reason is that a Lorentzian form needs the time coefficient to live in the opposite square class from the spatial coefficients, but if $c\in F_{p}$, then $c^{2}$ is a square. Thus, the correct time constant *c* is not available inside $F_{p}$; it exists in the next shell $F_{p^{2}}$, obtained by adjoining a square root of a chosen nonsquare $\nu \in F_{p}^{\times }$.

This formalizes (and sharpens) the FRC claim that “Minkowski emerges locally” only when one allows the minimal extension beyond the observer’s local algebraic horizon (compare also the “No South Pole in $F_{p}$” inaccessibility argument).

**Key contribution.**
  (i) A short nonexistence theorem: no $c\in F_{p}$ with $c^{2}=\nu$ for any nonsquare ν.
  (ii) A corollary: a genuine split-signature quadratic form requires $F_{p^{2}}$.
  (iii) A concrete p=13 example. All statements are elementary and reproducible.
**Scope and assumptions.** Throughout this note we restrict attention to symmetry-complete prime shells of order p=4t+1, as detailed in [1]. For such primes, −1 is a quadratic residue in $F_{p}$, and the element $i\in F_{p}$ with $i^{2}=-1$ exists, yielding the structural set {1,i,−1,−i} that underpins the rotational symmetry of each shell $S_{p}$. This condition is essential in the FRC framework, as it ensures the existence of a complete additive-multiplicative symmetry and the geometric interpretation of $S_{p}$ as a discrete two-sphere embedded in the symbolic symmetry space *U*.

For primes p≡3(mod4), −1 is a nonsquare and the element *i* does not exist in $F_{p}$; the internal Euclidean structure therefore breaks, and the shell is not symmetry-complete. Nevertheless, a Lorentzian analogue can still be constructed over the quadratic extension $F_{p^{2}}$, where *i* becomes available. For composite moduli q=4t+1 that are not prime, the FRC program generalizes the construction through the Chinese Remainder Theorem, producing a system of coupled prime subshells. These composite or non-symmetric cases lie beyond the minimal setting of the present technical note but are addressed in the broader FRC framework [1].

**Contextual framing.** In the broader FRC program, the emergence of a Lorentzian signature is not merely a technical algebraic choice but is tied to the reconstruction of causal structure itself. The distinction between Euclidean and Minkowski forms reflects whether time and space coordinates belong to the same or different square classes in the underlying finite field. When the time coefficient can only be realized in a quadratic extension, causality appears as a form of algebraic inaccessibility: it requires stepping “beyond the shell” of $F_{p}$. This connects directly with the horizon principles already identified in FRC (e.g., the inaccessibility of the South Pole in the orbital complex). The present note isolates this mechanism in a minimal form, showing that the Lorentzian split is impossible within a single prime shell and arises only in the extension, thereby grounding causal order in the square-class structure of finite fields.
**References and context.** The algebraic classification underlying our main theorem rests on the standard theory of quadratic forms over finite fields [5], where it is well known that of the nondegenerate forms in dimension at least three are isotropic and split into two equivalence classes distinguished by square classes of their coefficients; see Lam’s monograph [6] for a comprehensive treatment. On the physics side, our interpretation of the square-class obstruction as “algebraic causality” resonates with relational views of time and causality advocated by Smolin, who emphasizes that causal structure is not fundamental but emergent and relational [3]. This approach is also conceptually aligned with Noether’s foundational insight that every symmetry corresponds to a conservation law, here reinterpreted within finite algebraic structure where conservation and causality share a common discrete invariant [7]. Together these sources situate the present note both in the classical algebraic literature and in contemporary discussions of relational physics.

## 2. Quadratic Extension for Lorentzian Signature

**Preliminaries.** We recall the minimal algebraic facts needed here.

**Definition** **1** (Symmetry-complete shell; FRC notation)**.**
*Let p=4t+1 be prime. The multiplicative group $F_{p}^{\times }$ is cyclic of order 4t and contains the structural set {1,i,−1,−i} with $i^{2}=-1$. The ambient symmetry space U is formed from 4-tuples (t;a,m,ϵ) modulo the symmetry actions; the orbital complex $S_{p}\subset U$ is the 2D skeleton obtained by fixing ϵ=1, and combining additive meridians and multiplicative latitudes.*

**Euclidean vs. Lorentzian forms.** Over R, the Euclidean-Lorentzian dichotomy is determined by ordering: a Euclidean form
  $$Q_{E}\left(x,y,z\right)=x^{2}+y^{2}+z^{2}$$
  has all positive eigenvalues and represents the standard metric of three-dimensional space, whereas a Lorentzian form
  $$Q_{L}\left(x,y,z,t\right)=-c^{2}t^{2}+x^{2}+y^{2}+z^{2}$$
  has one negative and three positive eigenvalues—signature (1,3).

Over a finite field $F_{p}$ there is no total order compatible with field operations, since ordered fields have characteristic 0, and “sign” has no general meaning. Distinctions for quadratic forms are therefore purely algebraic, encoded by the two square classes of $F_{p}^{\times }$—squares vs. nonsquares—and the associated invariants: Witt index and discriminant [6]. More specifically, a diagonal quadratic form

(1) $$Q_{\nu }\left(t,x,y,z\right)=-\nu \;t^{2}+x^{2}+y^{2}+z^{2}$$

is called *Euclidean* if all diagonal coefficients lie in the same square class, and *Lorentzian* if the distinguished “time” coefficient −ν lies in the opposite square class to the three spatial coefficients. In the latter case the form is split with Witt index 1, which algebraically mirrors the real signature (1,3) without invoking order. See the finite-field formulation and usage in the present work and its algebraic context within the FRC program.

**Remark** **1** (Local correspondence near the frame origin)**.**
*For large primes p and coordinates bounded by local observer horizon $R\ll \sqrt{p}$, the integral quadratic form $Q_{\nu }\left(t,x,y,z\right)$ of Equation (1) over R and its reduction $Q_{\nu }\;\mathrm{mod}\;p$ coincide on the same integer lattice points. Consequently, in the R-neighbourhood of the frame-of-reference origin, the finite-field null set of $Q_{\nu }\;\mathrm{mod}\;p$ reproduces the real light cone $Q_{\nu }=0$ up to scaling. In this sense, the Lorentzian form defined over $F_{p}$ approaches the continuous Minkowski form over R as p grows. A complete proof of this correspondence, involving asymptotic density and the equidistribution of lattice points, lies beyond the scope of this note and is left for future work.*

We only use the following basic facts.
  (i) $F_{p}^{\times }$ splits into two square classes: the set of nonzero squares ${\left(F_{p}^{\times }\right)}^{2}$ and its complement (nonsquares). When p≡1(mod4), −1 is a square.
  (ii) If $c\in F_{p}$, then $c^{2}\in {\left(F_{p}^{\times }\right)}^{2}$ is a square.
  (iii) If $\nu \in F_{p}^{\times }$ is a nonsquare, the polynomial $X^{2}-\nu$ is irreducible over $F_{p}$ and defines the quadratic extension $F_{p^{2}}≃F_{p}\left[X\right]/\left(X^{2}-\nu \right)$.

**Lemma** **1** (Square-class absorption by coordinate rescaling)**.**
*Let $F_{p}$ be a finite field with p≡1(mod4). Consider a diagonal quadratic form*

$$Q\left(X\right)=a_{0}X_{0}^{2}+a_{1}X_{1}^{2}+\ldots +a_{n}X_{n}^{2}\;\left(n\geq 1\right),$$

*with each $a_{i}\in F_{p}^{\times }$. If $a_{i}/a_{0}$ is a square in $F_{p}^{\times }$ for all i, then Q is equivalent over $F_{p}$ to $a_{0}\;\left(X_{0}^{2}+\ldots +X_{n}^{2}\right)$. In particular, if −1 is a square (p≡1(mod4)), any common square class (all $a_{i}$ squares or all $a_{i}$ nonsquares) yields equivalence to a* Euclidean *positive-definite diagonal form.*

**Proof.**  Pick $u_{0}\in F_{p}^{\times }$ with $u_{0}^{2}=a_{0}$. For each *i*, choose $u_{i}\in F_{p}^{\times }$ with $u_{i}^{2}=a_{i}$ if $a_{i}$ is a square, and $u_{i}^{2}=\left(-1\right)a_{i}$ if $a_{i}$ is a nonsquare (possible since −1 is a square). Define $Y_{i}=\left(u_{i}/u_{0}\right)X_{i}$. Then $a_{i}X_{i}^{2}=a_{0}Y_{i}^{2}$ for all *i*, hence $Q\left(X\right)=a_{0}\sum_{i}Y_{i}^{2}$, proving the claim. □

**Main result.** The next theorem encodes the algebraic obstruction to Minkowski signature inside $F_{p}$.

**Theorem** **1** (Nonexistence of a causal square root in $F_{p}$)**.**
*Let p≡1(mod4), and let $\nu \in F_{p}^{\times }$ be a nonsquare. There is no $c\in F_{p}$ with $c^{2}=\nu$. Consequently, for any $c\in F_{p}$ the diagonal form*

$$Q\left(t,x,y,z\right)\;=\;-c^{2}\;t^{2}+x^{2}+y^{2}+z^{2}$$

*is equivalent over $F_{p}$ to a Euclidean diagonal form (all coefficients in one square class).*

**Proof.**  The first statement is tautological: $c^{2}$ is a square in $F_{p}^{\times }$, so it cannot equal a fixed nonsquare ν. For the consequence, since p≡1(mod4) we have $-1\in {\left(F_{p}^{\times }\right)}^{2}$, hence $-c^{2}$ is a square whenever $c^{2}$ is; thus all diagonal coefficients of *Q* lie in the same square class. Lemma 1 *Q* is equivalent to $a_{0}\;\left(T^{2}+X^{2}+Y^{2}+Z^{2}\right)$ for some $a_{0}\in F_{p}^{\times }$, i.e., a Euclidean form. See also Lam, *Introduction to Quadratic Forms over Fields* (classification over finite fields; isotropy in dimension ≥3; split vs. non-split by square classes) for background. □

**Corollary** **1** (Minimal quadratic extension for a Lorentzian split)**.**
*Fix a nonsquare $\nu \in F_{p}^{\times }$. The split-signature diagonal form*

$$Q_{\nu }\left(t,x,y,z\right)\;=\;-\nu \;t^{2}+x^{2}+y^{2}+z^{2}$$

*is not realizable over $F_{p}$ but is realized over the quadratic extension $F_{p^{2}}≅F_{p}\left[X\right]/\left(X^{2}-\nu \right)$ by adjoining $c:=X\;\;\;\mathrm{mod}\;(X^{2}-\nu )$ with $c^{2}=\nu$, so $Q_{\nu }=-c^{2}t^{2}+x^{2}+y^{2}+z^{2}$ has one coefficient in the square class opposite to the others (Witt index 1).*

**Proof.**  Non-realizability in $F_{p}$ follows from Theorem 1. In $F_{p^{2}}$ the element *c* with $c^{2}=\nu$ exists by construction, giving the desired split. Standard finite-field quadratic-form theory [6] identifies this as the split class with Witt index 1; see also the discussion and p=13 example in the present note. □

**Pointers to standard results.** Over finite fields of odd characteristic, nondegenerate quadratic forms in dimension ≥3 are isotropic, and diagonal forms are classified up to equivalence by dimension and discriminant; the Hasse invariant is trivial. The split vs. non-split dichotomy (Witt index) is detected by square classes of coefficients; see [6]. Our usage matches the finite-field notion of “Lorentzian”—one coefficient in the opposite square class—employed in this note and the broader FRC framework.

**Remark** **2** (Interpretation in FRC)**.**
*In the shell language, c does not exist as an internal element of $F_{p}$ if one insists that $c^{2}$ be a fixed nonsquare ν. Therefore, the* causal *split of square classes (time vs. space) requires a pass to the “next shell” $F_{p^{2}}$. This mirrors the horizon/inaccessibility interpretation in FRC exemplified by the “No South Pole in $F_{p}$” statement in [1].*

**Local Minkowski linearization.** FRC provides a framed-real embedding that supports local linearization around a frame point $\left(t;0,1,g\right)\in U_{t}$. In that calculus, once $Q_{\nu }$ is available (i.e., over $F_{p^{2}}$), one obtains a genuine local Minkowski quadratic form
  $$ds^{2}\;=\;-{\left(\lambda_{t}\;dt\right)}^{2}\;+\;{\left(\lambda_{1}\;dx\right)}^{2}+{\left(\lambda_{2}\;dy\right)}^{2}+{\left(\lambda_{3}\;dz\right)}^{2},$$
  for suitable positive calibrations $\lambda_{\mu }$ determined by the framed units. The proof is standard linearization: the discrete tangent and the symmetric bilinearization of $Q_{\nu }$ determine the form; the point is that the algebraic split of square classes needed for Lorentzian signature only exist after adjoining *c* (Corollary 1). All other steps are routine in the framed setup.

**Concrete example.** Take p=13. The nonzero squares and nonsquares are
  $${\left(F_{p}^{\times }\right)}^{2}=\left\{1,3,4,9,10,12\right\},\;\mathrm{nonsquares}=\left\{2,5,6,7,8,11\right\}.$$
  Hence no $c\in F_{p}$ satisfies $c^{2}\in \left\{2,5,6,7,8,11\right\}$. Pick ν=2. Then $X^{2}-2$ is irreducible over $F_{p}$, and
  $$F_{p^{2}}\;≃\;F_{p}\left[X\right]/\left(X^{2}-2\right),\;c:=X\;\mathrm{mod}\;\left(X^{2}-2\right),\;c^{2}=2.$$
  Thus
  $$Q_{2}\left(t,x,y,z\right)\;=\;-2\;t^{2}+x^{2}+y^{2}+z^{2}$$
  is realized over $F_{p^{2}}$ and provides the desired split. One can explicitly enumerate null solutions $Q_{2}=0$ in small boxes to visualize the (finite) light-cone counts; null sets exist in ≥3 variables over finite fields by standard isotropy arguments.

To illustrate how the Lorentzian structure established algebraically over $F_{p^{2}}$ can reproduce the familiar relativistic symmetries, we now formulate an explicit finite-field analogue of the Lorentz transformation that preserves the quadratic form $Q_{\nu }$.

## 3. Lorentz Transform

**Relative frames and boost parameter.** Let (t,0,1,g) denote a frame of reference in $S_{p}$, where *g* is a primitive root generating multiplicative symmetry. Two observers with generators $g_{1}=g^{m_{1}}$ and $g_{2}=g^{m_{2}}$ differ by a latitudinal step $\Delta m=m_{2}-m_{1}$. Define the *rapidity-like parameter*
  $$u:=g^{\Delta m}\in F_{p}^{\times }.$$

From *u* we construct
  $$\gamma =\frac{u+u^{-1}}{2}\in F_{p^{2}},\;a=\frac{u-u^{-1}}{2c}\in F_{p^{2}}.$$

These satisfy the identity
  $$\gamma^{2}-\nu a^{2}=1,$$
  ensuring preservation of $Q_{\nu }$.

**Lorentz transformations in 1 + 1 dimensions.** Define the 2×2 matrix
  $$\Lambda \left(u\right)=\left(\begin{array}{cc} \gamma & a\\ \nu a & \gamma \end{array}\right).$$

Then for vectors ${\left(t,x\right)}^{⊤}\in F_{p^{2}}^{2}$,
  $$\left(\begin{array}{c} t^{\prime }\\ x^{\prime } \end{array}\right)=\Lambda \left(u\right)\left(\begin{array}{c} t\\ x \end{array}\right)$$
  satisfies
  $$-\nu t^{\prime 2}+x^{\prime 2}=-\nu t^{2}+x^{2}.$$

Thus $\Lambda \left(u\right)\in O\left(Q_{\nu }\right)$ is a *finite Lorentz boost*.

**Velocity, β, and Lorentz factor.** The boosted spatial axis $x^{\prime }=0$ implies
  $$\nu a\;t+\gamma x=0\;\Rightarrow \;v=\frac{dx}{dt}=-\;\frac{\nu a}{\gamma }.$$

The dimensionless velocity is
  $$\beta =\frac{v}{c}=-\;\frac{ca}{\gamma }=-\frac{u-u^{-1}}{u+u^{-1}}.$$

From $\gamma^{2}-{\left(ca\right)}^{2}=1$ it follows that
  $$\gamma =\frac{1}{\sqrt{1-\beta^{2}}}.$$

Thus γ and β are determined exactly from $u=g^{\Delta m}$.

**Velocity addition law.** Since $\Lambda \left(u_{1}\right)\Lambda \left(u_{2}\right)=\Lambda \left(u_{1}u_{2}\right)$, the parameter *u* composes multiplicatively. Equivalently, the velocities compose by the Einstein addition law
  $$v_{12}=\frac{v_{1}+v_{2}}{1+v_{1}v_{2}/c^{2}}.$$

**Extension to 1 + 3 dimensions.** For a boost along the *x*-axis, one obtains
  $$\Lambda_{x}\left(u\right)=\left(\begin{array}{cccc} \gamma & -\gamma v/\nu & 0 & 0\\ -\gamma v & \gamma & 0 & 0\\ 0 & 0 & 1 & 0\\ 0 & 0 & 0 & 1 \end{array}\right),$$
  with $y^{\prime },z^{\prime }$ unchanged. Similar constructions apply for boosts along *y* or *z*, and rotations in $O(3,F_{p})$ combine to yield the full Lorentz group $O(1,3;F_{p^{2}})$.

## 4. Discussion and Outlook

The explicit realization of the Minkowski quadratic form and its associated Lorentz group within the finite-field framework provides a comprehensive justification for the use of the term *Relativistic Algebra* in the broader FRC program. Together, these constructions demonstrate that the essential structural features of special relativity—the invariant space-time interval and the symmetry group preserving it—emerge naturally and rigorously from the algebraic properties of finite fields and their quadratic extensions. In this sense, the FRC framework embodies relativity not as an external postulate but as an intrinsic algebraic property of finite arithmetic symmetries. Such physical interpretations pave the way for the development of finite-field analogues of relativistic physics, potentially offering new insights into the discrete and informational foundations of space-time, which we refer to as *Finite Ring Cosmology*.

Theorem 1 isolates the minimal algebraic reason why a single space-like prime shell does not carry Lorentzian geometry: the time coefficient must belong to a nonsquare class, which forbids its realization as $c^{2}$ with $c\in F_{p}$. Corollary 1 shows that the quadratic extension $F_{p^{2}}$ is sufficient (and minimal) to restore the split, giving a precise sense in which causality emerges “one shell out”. Specifically, the quadratic form

$$Q_{\nu }\left(t,x,y,z\right)=-\;\nu \;t^{2}+x^{2}+y^{2}+z^{2}$$

defined over $F_{p^{2}}$ admits a nondegenerate symmetric bilinear form $B_{\nu }\left(u,v\right)=\frac{1}{2}\left(Q_{\nu }\left(u+v\right)-Q_{\nu }\left(u\right)-Q_{\nu }\left(v\right)\right)$. The group of linear automorphisms preserving this form,

$$O\left(Q_{\nu },F_{p^{2}}\right)=\left\{\;A\in {\mathrm{GL}}_{4}\left(F_{p^{2}}\right)|Q_{\nu }\left(Ax\right)=Q_{\nu }\left(x\right)\;\right\},$$

is a finite orthogonal group of split type (Witt index 1) in dimension 4, sometimes denoted $O_{4}^{+}\left(F_{p^{2}}\right)$ in the literature on finite classical groups [8,9]. This group plays the role of a Lorentz group in the finite-field setting: it preserves the Lorentzian quadratic form exactly, though it is a finite algebraic group rather than a continuous group O(1,3) over R. Accordingly, our earlier phrasing has been replaced by the precise statement that a nontrivial finite orthogonal group $O(Q_{\nu },F_{p^{2}})$ of split type acts on the Lorentzian form $Q_{\nu }$.

The algebraic obstruction identified above—that a Lorentzian split requires a quadratic extension—can be interpreted heuristically as the emergence of causal separation. Within a single prime shell $F_{p}$, the coefficient distinguishing time from space is algebraically inaccessible; it exists only in the space-time quadratic extension $F_{p^{2}}$. In this sense, causality appears as algebraic inaccessibility. From the viewpoint of a finite observer with a bounded informational horizon, the successive enlargement of accessible symmetries across shells manifests as an apparent growth of entropy, offering a discrete algebraic analogue of the Second Law of Thermodynamics. These heuristic interpretations are conceptual and not yet asserted as formal theorems. They align with the finite and relational perspectives of Lev [2] and Smolin [3] and are intended to motivate further study rather than to claim a definitive correspondence.

In FRC, the succession of shells indexed by the chronon parameter t constitutes a discrete informational timeline: each new shell $S_{q}$ expands the available algebraic symmetries and square-class distinctions, thereby enlarging the catalogue of accessible states. Cosmic time, in this view, quantifies the accumulation of structural information: the higher the radius t, the greater the informational complexity encoded in the symmetry shell $S_{q}$. Entropy, in this finite setting, reflects the mismatch between the observer’s bounded horizon and the build-up of relational complexity in the symmetry shells, with causality and the arrow of time emerging as two facets of the same algebraic phenomenon governed by square-class structure.

**Related work.** Square/nonsquare classes and quadratic extensions are textbook facts in finite-field theory and underlie quadratic-form classification over finite fields [6,8,9]. The FRC-specific notions of shells $U_{t}$, the orbital complex $S_{p}$, framed numbers, and the horizon/inaccessibility perspective (e.g., “No South Pole in $F_{p}$”) are taken from [1]. The novelty here lies in the causal interpretation: split signature is equivalent to a square-class separation unattainable inside $F_{p^{2}}$ but realized in the minimal extension, thus tying causality to “next-shell” accessibility.

**Outlook.** The algebraic obstruction we have identified has immediate consequences for how causal structure may be represented in finite settings. The passage to $F_{p^{2}}$ that restores the Lorentzian split also yields nontrivial null sets $Q_{\nu }=0$, which can be viewed as discrete analogues of light-cone structures. This suggests that finite-field shells equipped with square-class-separated quadratic forms could serve as models for spacetime in discrete or algebraic approaches to physics. Possible applications include finite-field analogues of Minkowski space in coding theory and simplified models for causal order in discrete quantum-gravity frameworks. In this sense, algebraic causality not only explains the emergence of Lorentzian signature in FRC but also provides a bridge toward broader investigations of finite geometries of space-time, including the formulation of Schrödinger-Dirac dynamics within the same finite-field framework, which we develop in the forthcoming work [10].

## Data Availability

All data is contained within the article.
